# The long-term outcomes of posterior nasal neurectomy with or without pharyngeal neurectomy in patients with allergic rhinitis: a randomized controlled trial

**DOI:** 10.1016/j.bjorl.2021.05.006

**Published:** 2021-05-29

**Authors:** Hongting Hua, Guoyuan Wang, Yi Zhao, Dong Wang, Zengyu Qiu, Ping Fang

**Affiliations:** First Affiliated Hospital of Anhui Medical University, Department of Otorhinolaryngology Head and Neck Surgery, Hefei, Anhui, China

**Keywords:** Allergic rhinitis, Posterior nasal neurectomy, Pharyngeal branches of the pterygopalatine ganglion, Chronic cough, Asthma

## Abstract

**Introduction:**

Allergic rhinitis is a form of IgE mediated inflammation of the nasal mucosa in response to specific allergens, resulting in typical symptoms.

**Objectives:**

This study was designed with the primary goal of comparing the clinical efficacy of posterior nasal neurectomy with or without pharyngeal neurectomy for the treatment of moderate-to-severe perennial allergic rhinitis. Secondary study aims included a comparison of the severity of comorbidities, including chronic cough and asthma, between patients in these two surgical treatment groups.

**Methods:**

A total of 52 patients were enrolled in this randomized controlled trial and were assigned to either the control group (posterior nasal neurectomy) or the experimental group (posterior nasal neurectomy + pharyngeal neurectomy). The visual analog scale and rhinoconjunctivitis quality of life questionnaire were used to compare the differences in patient symptoms between baseline and 6-, 12-, and 24-months post-treatment. In addition, patient cough and asthma symptoms were monitored during follow-up via visual analog scale and asthma control test respectively.

**Results:**

No significant differences in preoperative scores were evident between groups (*p* > 0.05). At 6-months post-treatment, there were significant differences in visual analog scale, rhinoconjunctivitis quality of life questionnaire, and asthma control test scores relative to baseline values in experimental group and control group patients (*p* < 0.05), and this remained true upon 12- and 24-month follow-up. No significant differences in visual analog scale, rhinoconjunctivitis quality of life questionnaire, or asthma control test scores were observed between the two treatment groups at any postoperative follow-up time point (*p* > 0.05), while coughing severity was found to be significantly reduced in the experimental group relative to the control group (*p* < 0.05).

**Conclusion:**

posterior nasal neurectomy can be safely implemented with or without pharyngeal neurectomy in order to effectively treat allergic rhinitis. Combined posterior nasal neurectomy and pharyngeal neurectomy treatment may offer greater value than posterior nasal neurectomy alone for the treatment of allergic rhinitis patients with chronic cough.

## Highlights

• The long-term outcomes of posterior nasal neurectomy in patients with allergic rhinitis.
• A RCT comparing the clinical efficacy of posterior nasal neurectomy with or without pharyngeal neurectomy.
• Posterior nasal neurectomy combined with pharyngeal neurectomy for allergic rhinitis patients with chronic cough.

## Introduction

Allergic rhinitis (AR) is a form of immunoglobulin E (IgE) mediated inflammation of the nasal mucosa in response to specific allergens, resulting in symptoms including itching, nasal obstruction, watery rhinorrhea, and sneezing in the absence of infection.^1^, ^2^ AR patients may also suffer from ocular symptoms, chronic cough, and postnasal drip, significantly reducing the quality of life in a subset of affected individuals.^1^ The first-line treatment of AR typically consists of the administration of second-generation non-sedative antihistamines together with intranasal steroids. In patients who fail to exhibit satisfactory responses to such treatment, subcutaneous or sublingual allergen-specific immunotherapy can often effectively alleviate symptoms.^2^ However, a subset of patients is refractory to these conservative interventions, and in these cases, surgical treatments including vidian neurectomy or posterior nasal neurectomy (PNN) represent viable alternative treatments.^3^

**Figure 1:**
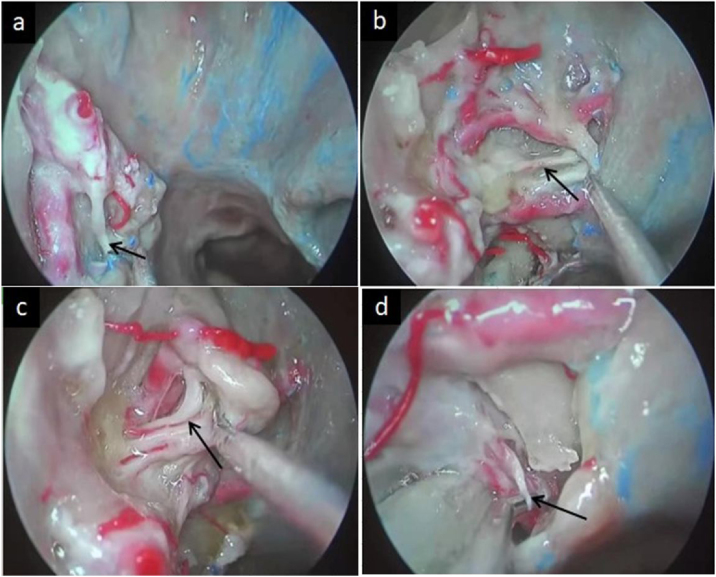
The posterior nasal nerve emerges from the sphenopalatine foramen and is distributed to the nasal mucosa following the branches of the sphenopalatine vessels. (a) The anteroinferior branch of the posterior nasal nerve toward the inferior turbinate. (b) The branch of the posterior nasal nerve toward the nasal septum. (c) The posterosuperior branch of the posterior nasal nerve toward the superior turbinate. (d) The pharyngeal nerve transmitted by the palatovaginal canal (PVC).

The posterior nasal nerve is a peripheral branch of the sphenopalatine ganglion containing multiple groups of small yet independent nerve fascicles that passes into the nasal cavity via the sphenopalatine foramen (Fig. 1a–c), and that is composed of postganglionic pterygoid nerve and maxillary nerve sensory fibers.^4^, ^5^ PNN is a form of highly selective vidian neurectomy that can effectively treat severe AR by limiting nasal mucosal hypersensitivity and suppressing associated secretory activity.^3^, ^6^, ^7^ PNN also minimizes the risk of irreversible complications of vidian neurectomy, including palatal numbness and persistent dry eye.^5^

The nasopharyngeal mucosa is innervated by the pharyngeal nerve, which is an additional branch of the pterygopalatine ganglion transmitted by the palatovaginal canal (PVC) (Fig. 1d), and that is continuous with the region of the nasal mucosa regulated by the posterior nasal nerve.^8^ Given this functional similarity and proximity, we hypothesized that the combined resection of both the posterior nasal nerve and the pharyngeal nerve may result in altered clinical outcomes in AR patients as compared to conventional PNN.

No prior studies have evaluated the outcomes of pharyngeal neurectomy (PN) in AR patients. We therefore designed the present trial to compare the clinical efficacy of combination PNN and PN to that of PNN alone in AR patients by monitoring long-term symptom control and the severity of cough and asthma in treated individuals.

## Patients and methods

### Patients

Between February 2016 and February 2019, 52 patients with moderate-to-severe retractable perennial AR were enrolled in the present study, which received ethical approval from The Committee on Medical Ethics of our hospital (Reference nº Quick-PJ 2020-13-15). All participants signed an Informed Consent document prior to participation. Study inclusion criteria were: 1) All patients were diagnosed with AR as per the Chinese Society of Allergy Guidelines for Diagnosis and Treatment of Allergic Rhinitis,^1^ which define AR based upon signs, symptoms, and allergen detection; 2) All patients had either failed to achieve satisfactory outcomes following treatment with standard pharmaceutical regimens or were unable to tolerate these treatments or specific immunotherapy.

Patients were excluded from participating in the present study if they met the following criteria: 1) patients who had not previously been treated with standard pharmaceutical regimens or via specific immunotherapy; 2) patients that had concurrent asthma that was not well controlled; 3) patients with coagulation disorders; 4) patients unable to tolerate surgical treatment; 5) patients with acute infections or fevers immediately prior to surgery; 6) patients with documental mental disorders or poor compliance; 7) patients not able to accept the potential complications and risks associated with surgical treatment; 8) patients with a history of prior nasal nerve surgery; 9) patients with evidence of acute/chronic sinusitis, nasal polyps, nasal cysts, or any cancers of the nasal cavity or sinuses upon sinonasal computed tomography (CT) assessment; and 10) patients with a history of smoking.

### Randomization

**Figure 2:**
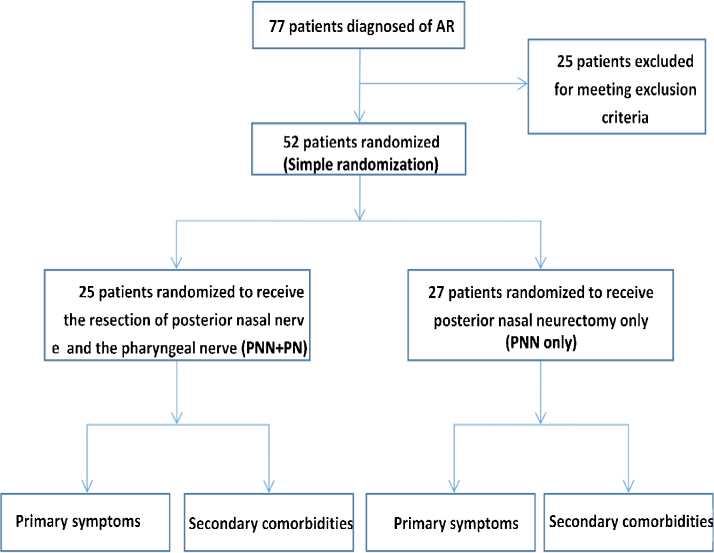
Study flowchart.

The 52 patients enrolled in this study had been diagnosed with AR for 1–10 years at the time of diagnosis and were randomly assigned to either an experimental group that underwent PNN and PN or a control group that underwent PNN alone through simple randomization. The flowchart for this study is shown in Fig. 2. Patients in both groups suffering from nasal obstruction as a result of the deviation of the nasal septum or inferior turbinate hypertrophy also underwent combination of septoplasty and/or submucosal inferior turbinectomy.

### Preoperative evaluation

Patients enrolled in the present study were not treated with any specific anti-allergenic medications or via specific immunotherapy within 30 days prior to surgical treatment. Preoperative endoscopic examinations and sinonasal CT scans were conducted in all patients for the purposes of baseline evaluation. In patients with comorbid asthma, pulmonary function tests were additionally conducted in the respiratory department of our institution, and appropriate treatments were provided to alleviate abnormal lung function in all patients with abnormal preoperative lung functionality.

### Surgical intervention

**Figure 3:**
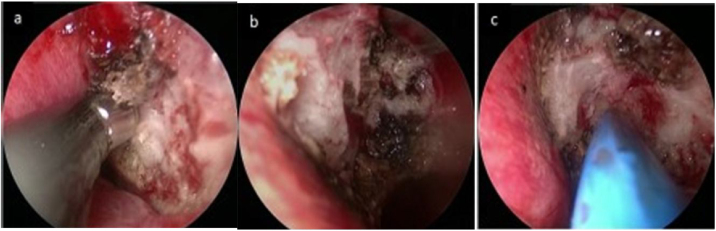
(a) Endoscopic view showing the sphenopalatine foramen. (b) Resection of the anteroinferior branch of the posterior nasal nerve. (c) Resection of the pharyngeal nerve from the palatovaginal canal.

All surgical procedures in the present study were conducted by the same surgeon and were performed under general anesthesia using a 0° nasal endoscope with a 4 mm bilateral diameter (Karl Storz). For PNN, a vertical incision was made in the posterior portion of the middle nasal turbinate. Dissection was performed along the bone to elevate the mucosal flap to expose the thick orbital process of the vertical plate of the palatine bone and the sphenopalatine foramen. The postganglionic pterygopalatine rami run along with the branches of sphenopalatine artery (SPA) forming the neurovascular fascicles through the sphenopalatine foramen. The 3–4 mm of mucosal and submucosal neurovascular bundles surrounding the sphenopalatine foramen were subjected to full radiofrequency ablation, directly to the bone. (Fig. 3a–b). For PN, the sphenoid process of the palatine bone was then removed from the posterior portion of the sphenopalatine foramen to expose the neurovascular bundle in the palatovaginal canal, and the pharyngeal nerve was completely electrocoagulated (Fig. 3c). Septoplasty and inferior turbinatoplasty were performed as appropriate in individual patients, as discussed above. A gelatin sponge was then inserted into the middle nasal meatus to complete the operation.

For 1–3 months postoperatively, patients were administered a nasal steroid spray and conducted daily nasal irrigation, with the duration of these postoperative treatments being dependent upon symptom severity and mucosal conditions. When necessary, nasal endoscopy was additionally performed.

### Evaluation

Changes in symptom severity, quality of life, and secondary comorbidity severity (cough and asthma) were evaluated in all patients by comparing pre- and postoperative symptoms according to patient self-assessment scores. The severity of symptoms including rhinorrhea, nasal obstruction, sneezing, and nasal itching was evaluated with a visual analog scale (VAS), with individual symptoms being scores from 0 to 10 (0 = no symptoms; 10 = most severe discomfort). In addition, patients completed the rhinoconjunctivitis quality of life questionnaire (RQLQ) both before and after surgery in order to compare the quality of life within and between groups. This questionnaire is composed of 28 questions assessing 7 domains (sleep, non-nasal/eye symptoms, emotional function, practical problems, nasal symptoms, eye symptoms, and activities). All symptoms were scored from 0 to 6, with 0 corresponding to no symptoms and 6 corresponding to the most severe discomfort. These domains were further subdivided into four groups: nasal/eye symptoms (NES), non-nasal/eye symptoms (NNES), sleep disorders, and others (including the practical problem, activity, and emotional function domains). Asthma control was assessed in those patients previously diagnosed with asthma, using an asthma control test (ACT), while cough severity was quantified using a VAS.

### Follow-up

Patients underwent regular in-person clinical follow-up at 6-, 12-, and 24-months post-surgery. During each follow-up visit, VAS and RQLQ questionnaires were completed, and changes in secondary comorbidities were evaluated. In addition, any complications that arose during the postoperative period were recorded.

### Data statistics

SPSS v22.0 for Windows (IBM Corp., NY, USA) was used for all statistical analyses in the present study. The normality of these data was evaluated via the Shapiro–Wilk test. Continuous data are given as means ± Standard Deviation (SD). For normally distributed data, values between the experimental and control groups were compared via independent sample *t*-tests, whereas changes in values over time within groups were compared via matched sample *t*-tests. For non-normally distributed data, values were instead compared via the Wilcoxon signed-rank test. Chi-Squared tests were used to compare categorical variables; p < 0.05 was the significance threshold.

## Results

### Baseline patient characteristics

In total, 77 patients were screened for eligibility for the present trial, of whom 25 were excluded in accordance with study exclusion criteria. The remaining 52 patients were randomized into either the experimental group (PNN + PN, n = 25) or the control group (PNN, n = 27). All patients completed the VAS and RQLQ questionnaires at baseline and at 6 months postoperatively, while 48 patients completed these questionnaires at 12 months postoperatively (23 in the experimental group and 25 in the control group; 2 per group were lost to follow-up), and 41 patients completed these questionnaires at 24 months postoperatively (20 patients in the experimental group and 21 patients in the control group, with remaining patients having been lost to follow-up or unable to complete the follow-up period). The baseline demographic characteristics and clinical findings in study patients are compiled in Table 1. There were no significant differences between the experimental and control groups with respect to patient age, gender, AR duration, or preoperative VAS or RQLQ scores (Table 1) (*p* > 0.05). There were also roughly equal rates of comorbid nasal septum deviation and inferior turbinate hypertrophy in these two groups. Baseline ACT scores also indicated that the presence and severity of comorbid asthma did not differ significantly between groups (16.11 ± 1.90 vs. 16.60 ± 2.22, p > 0.05).

**Table 1:** Baseline demographic characteristics and clinical findings in study patients.

Variables	PNN + PN	PNN	*p*-Value
Number of patients	25	27	–
Gender (male/female)	16/9	15/12	0.535
Age (years, mean ± SD)	37.04 ± 8.41	36.44 ± 7.71	0.791
Duration of AR (years, mean ± SD)	5.76 ± 2.47	6.56 ± 1.89	0.196
**VAS** (mean ± SD)			
Rhinorrhea	7.24 ± 1.20	7.07 ± 1.33	0.693
Nasal obstruction	6.52 ± 1.48	6.70 ± 1.38	0.645
Sneezing	7.00 ± 1.26	6.96 ± 1.26	0.916
Nasal itching	6.28 ± 1.75	6.41 ± 1.42	0.773
**RQLQ** (mean ± SD)			
NES	20.48 ± 3.16	20.30 ± 3.60	0.846
NNES	14.68 ± 3.40	15.30 ± 4.24	0.568
Sleep disorders	7.08 ± 2.20	7.63 ± 2.59	0.415
Others	24.88 ± 5.05	24.26 ± 5.16	0.663
**Comorbidity**			
Cough (VAS score, mean ± SD)	3.68 ± 1.77	3.81 ± 1.59	0.774
Asthma (number of patients)	9	10	0.938
Asthma (ACT score, mean ± SD)	16.11 ± 1.90	16.60 ± 2.22	0.615
Deviated nasal septum (number of patients)	12	16	0.416
Hypertrophy of inferior turbinate (number of patients)	6	7	0.873

PNN, posterior nasal neurectomy; PN, pharyngeal neurectomy; AR, allergic rrhinitis; SD, standard deviation; VAS, visual analog scale; RQLQ, rhinoconjunctivitis quality of life questionnaire; NES, nasal/eye symptoms; NNES, non-nasal/eye symptoms; ACT, asthma control test.

### Postoperative changes in primary symptom severity

**Figure 4:**
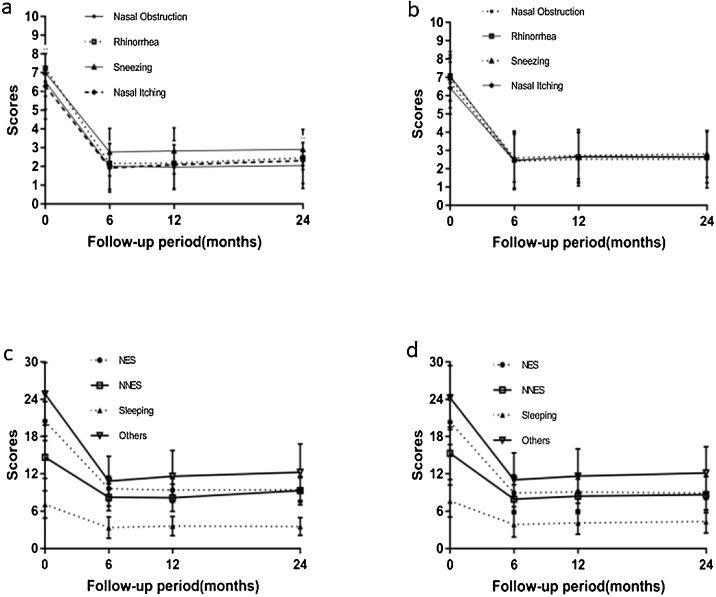
Changes in VAS and RQLQ scores in the experimental group and the control group. (a) Changes in VAS scores in the PNN + PN group. (b) Changes in VAS scores in the PNN group. (c) Changes in RQLQ scores in the PNN + PN group. (d) Changes in RQLQ scores in the PNN group. PNN, posterior nasal neurectomy; PN, pharyngeal neurectomy; VAS, visual analog scale; RQLQ, rhinoconjunctivitis quality of life questionnaire; NES, nasal/eye symptoms; NNES, non-nasal/eye symptoms.

Mean VAS and RQLQ scores for patients in both study groups during the follow-up period are compiled in Tables 2, 3 and Fig. 4. In the experimental group, we detected significant differences in VAS and RQLQ scores at 6 months post-treatment relative to baseline values (*p* < 0.05), and these values did not change further upon 12- or 24-month follow-up (Table 2, Table 3). The same trend was also observed in the control group.

**Table 2:** Mean VAS scores in the experimental and control groups.

	PNN + PN	PNN	*p*-Value
**Rhinorrhea** (mean ± SD)			
Preoperative	7.24 ± 1.20	7.07 ± 1.33	0.639
0.5 year	2.16 ± 1.14^a^	2.48 ± 1.58^a^	0.402
1 year	2.17 ± 1.11^a^	2.64 ± 1.35^a^	0.201
2 years	2.45 ± 1.15^a^	2.62 ± 1.40^a^	0.675
**Nasal obstruction** (mean ± SD)			
Preoperative	6.52 ± 1.48	6.70 ± 1.38	0.645
0.5 year	2.00 ± 1.23^a^	2.41 ± 1.55^a^	0.301
1 year	1.96 ± 1.87^a^	2.52 ± 1.65^a^	0.149
2 years	2.05 ± 1.23^a^	2.52 ± 1.57^a^	0.291
**Sneezing** (mean ± SD)			
Preoperative	7.00 ± 1.26	6.96 ± 1.26	0.916
0.5 year	2.76 ± 1.27^a^	2.59 ± 1.31^a^	0.642
1 year	2.83 ± 1.23^a^	2.72 ± 1.28^a^	0.771
2 years	2.90 ± 1.07^a^	2.81 ± 1.29^a^	0.809
**Nasal itching** (mean ± SD)			
Preoperative	6.28 ± 1.75	6.41 ± 1.42	0.773
0.5 year	1.92 ± 1.29^a^	2.44 ± 1.50^a^	0.184
1 year	2.09 ± 1.28^a^	2.68 ± 1.46^a^	0.143
2 years	2.30 ± 1.22^a^	2.67 ± 1.43^a^	0.383

VAS, visual analog scale; SD, standard deviation; PNN, posterior nasal neurectomy; PN, pharyngeal neurectomy.

The student’s *t*-test was used for statistical analysis.

a *p* < 0.05 vs. preoperative.

**Table 3:** Mean RQLQ scores in the experimental and control groups.

	PNN + PN	PNN	*p*-Value
**NES** (mean ± SD)			
Preoperative	20.48 ± 3.16	20.30 ± 3.60	0.846
0.5 year	9.64 ± 1.98^a^	8.93 ± 2.87^a^	0.298
1 year	9.39 ± 1.85^a^	9.08 ± 2.91^a^	0.658
2 years	9.45 ± 1.96^a^	8.90 ± 2.70^a^	0.466
**NNES** (mean ± SD)			
Preoperative	14.68 ± 3.40	15.30 ± 4.24	0.568
0.5 year	8.24 ± 2.19^a^	7.93 ± 2.30^a^	0.617
1 year	8.17 ± 2.1^a^	8.40 ± 2.75^a^	0.756
2 years	9.30 ± 2.30^a^	8.67 ± 2.90^a^	0.445
**Sleeping** (mean ± SD)			
Preoperative	7.08 ± 2.20	7.63 ± 2.59	0.415
0.5 year	3.36 ± 1.73^a^	3.85 ± 2.01^a^	0.351
1 year	3.61 ± 1.56^a^	4.12 ± 1.83^a^	0.305
2 years	3.55 ± 1.03^a^	4.33 ± 1.85^a^	0.139
**Others** (mean ± SD)			
Preoperative	24.88 ± 5.05	24.26 ± 5.16	0.663
0.5 year	10.84 ± 3.99^a^	11.04 ± 4.33^a^	0.865
1 year	11.61 ± 4.18^a^	11.64 ± 4.38^a^	0.980
2 years	12.25 ± 4.56^a^	12.14 ± 4.23^a^	0.938

RQLQ, rhinoconjunctivitis quality of life questionnaire; SD, standard deviation; PNN, posterior nasal neurectomy; PN, pharyngeal neurectomy; NES, nasal/eye symptoms; NNES, non-nasal/eye symptoms.

The student’s *t*-test was also used for statistical analysis.

a *p* < 0.05 vs. preoperative.

We did not detect any significant differences in preoperative VAS or RQLQ scores between groups (*p* > 0.05). We also observed no significant differences between groups with respect to postoperative symptoms or quality of life at any analyzed time point (*p* > 0.05).

### Changes in postoperative comorbidities

We additionally evaluated chronic cough and asthma incidence and severity in patients using VAS and ACT scales (Table 4). We detected no significant differences in these values at baseline between groups (*p* > 0.05), and we found that both comorbidities were significantly ameliorated at all postoperative time points in both groups (*p* < 0.05). However, we found that control of chronic cough was better in the experimental group relative to the control group at all three postoperative follow-up time points (*p* < 0.05), whereas asthma control did not differ significantly between these groups at any follow-up time point (*p* > 0.05).

**Table 4:** Comparisons of comorbidities in the experimental and control groups.

	Chronic cough (mean ± SD)			ACT (mean ± SD)		
	PNN + PN	PNN	*p*-value	PNN + PN	PNN	*p*-value
Preoperative	3.68 ± 1.77	3.81 ± 1.59	0.774	16.11 ± 1.90^a^	16.60 ± 2.22^a^	0.615
0.5 year	1.76 ± 1.05^a^	2.52 ± 1.50^a^	0.042^b^	19.89 ± 1.45^a^	20.10 ± 1.66^a^	0.773
1 year	1.74 ± 1.03^a^	2.60 ± 1.53^a^	0.033^b^	19.33 ± 0.71^a^	19.30 ± 0.40^a^	0.968
2 years	1.65 ± 1.09^a^	2.62 ± 1.66^a^	0.033^b^	19.22 ± 1.39^a^	19.60 ± 2.07^a^	0.650

ACT, asthma control test; SD, standard deviation; PNN, posterior nasal neurectomy; PN, pharyngeal neurectomy.

The student’s *t*-test was used for statistical analysis again.

a p < 0.05 vs. preoperative.

b p < 0.05 between PNN + PN group and PNN group.

### Complications and mortality

All patients were discharged roughly three days post-surgery, with no incidence of perioperative mortality. There were no major postoperative complications such as orbital or cranial nerve injury, massive nasal bleeding, palatal numbness, or persistent dry eye. One patient in the experimental group suffered from unilateral nasal hemorrhage one day after the operation, and a site of bleeding on the lateral wall of the right nasal cavity was found upon endoscopic examination. He recovered after we performed electrocoagulation of this wound, with intranasal packing with gelatin sponges having been performed under local anesthesia in the endoscopy room rather than returning to the operation room. There was no evidence of crusting or nasal synechiae in either nasal cavity.

## Discussion

Previous studies have shown that the posterior nasal nerve is composed of multiple individual postganglionic rami which supply the nasal mucosa, and selectively transecting these nerve fascicles in AR patients can achieve a similar therapeutic effect to that of vidian neurectomy.^4^ Our data suggest that the minimally invasive PNN procedure can significantly alleviate primary AR symptoms without causing significant complications associated with vidian neurectomy. Combined PNN and PN treatment did not significantly alter AR patient quality of life or symptoms relative to PNN alone. It is important to note that PNN may be associated with higher rates of long-term recurrence as compared to traditional vidian neurectomy as a consequence of resected nerve regeneration, as has been observed in preclinical studies, or due to a failure to fully resect all branches of the posterior nasal nerve during surgery.^9^, ^10^, ^11^ The exploration of more precise and reliable surgical procedures for the treatment of AR patients thus remains important.

Epidemiological studies suggest that asthma and AR often coexist and that there are similarities in the mechanisms governing the development of these two conditions.^12^ Given this overlap, the surgical treatment of AR has the theoretical potential to benefit asthma control. Consistent with such a hypothesis, we found that PNN conducted alone or in combination with PN was sufficient to significantly improve asthma control in AR patients, with no significant differences in the degree of control between these two procedures. Ai et al. similarly concluded that bilateral endoscopic vidian neurectomy was sufficient to improve both AR symptoms and asthma control in patients affected by both of these conditions.^13^ Vidian neurectomy or PNN may therefore be optimal approaches to controlling asthma in AR patients with comorbid asthma.

We also found that PNN conducted with or without PN was sufficient to alleviate chronic cough in AR patients in the present study. Non-asthmatic eosinophilic bronchitis (NAEB), upper airway cough syndrome (UACS), and asthma (cough variant asthma, CVA) are the most common drivers of chronic cough.^14^ Liu et al. previously found that in AR patients, chronic cough is caused by UACS, NAEB, CVA, and gastroesophageal reflux in 25.6%, 27.5%, 24.7%, and 5.1% of patients, respectively.^15^ We found that chronic cough improved significantly in patients after surgery, but we did not evaluate the etiology of chronic cough in these patients. The fact that surgery improved chronic cough in these patients may be attributable to reductions in postoperative nasal secretions, thereby resulting in reduced stimulation of the hyperresponsive cough reflex in these patients following neurectomy.^16^ Moreover, as asthma was improved by neurectomy in these patients, asthma-associated coughs were likely to be similarly improved. Upper airway obstruction can also increase intrathoracic pressure and thereby increase the odds of laryngopharyngeal and gastroesophageal reflux. Alleviating nasal obstruction can reduce the pressure of continuous positive airway pressure devices, resulting in the postoperative alleviation of gastroesophageal reflux-related cough.^17^

Interestingly, we found that chronic cough scores declined more significantly in the PNN + PN treatment group relative to the PNN treatment group. The reasons for this difference are unclear. One possibility is that PN resulted in further reductions in nasopharyngeal mucosal hypersensitivity to physical and chemical stimulation, consistent with the mechanistic basis for PNN-mediated alleviation of AR. Combination PNN and PN treatment may therefore be an optimal approach to treating AR patients suffering from a chronic cough.

PNN can avoid many of the complications of traditional vidian neurectomy, but the risk of significant nasal bleeding is still a remarkable risk that can result from SPA injury.^9^, ^18^ when we conducted PNN, we therefore retained the trunk of the SPA and prevented it from retracting into the sphenopalatine foramina to avoid severe nasal bleeding during and after surgery. The PVC transmits the pharyngeal nerve and the pharyngeal artery from the posterior wall of the pterygopalatine fossa to the nasopharyngeal roof and is proximal to the vidian canal.^8^, ^19^ The vidian nerve must be protected from damage when conducting PN in order to avoid causing complications including palatal numbness and persistent dry eye.

This study has a number of limitations that must be considered when interpreting our results. For one, this was a single-center study with limited sample size. In addition, VAS and RQLQ scores were subjective. Furthermore, chronic cough etiology was not explored in enrolled patients. Future large-scale studies using objective outcome measurements are therefore necessary to firmly establish the relative utility of PNN with or without PN for the alleviation of AR symptoms and associated comorbidities. In addition, accurate etiological analyses of patients with comorbid chronic coughs will be important in such studies.

## Conclusions

Our results demonstrate that PNN conducted with or without PN can achieve similar improvements in postoperative symptoms, indicating that both of these approaches are safe and effective for AR patient treatment. In AR patients suffering from pronounced chronic cough, combination PNN and PN treatment is a better option, whereas PNN is recommended as a first-line treatment in AR patients without severe chronic cough.

## Conflicts of interest

The authors declare no conflicts of interest.
